# Improving capacity for phytoremediation of Vetiver grass and Indian mustard in heavy metal (Al and Mn) contaminated water through the application of clay minerals

**DOI:** 10.1007/s11356-023-26083-5

**Published:** 2023-03-02

**Authors:** Beatrice Omonike Otunola, Makhosazana P. Aghoghovwia, Melusi Thwala, Alba Gómez-Arias, Rian Jordaan, Julio Castillo Hernandez, Olusola Oluwayemisi Ololade

**Affiliations:** 1 Centre for Environmental Management, University of the Free State, PO Box 339, Bloemfontein, 9300 South Africa; 2 Department of Soil, Crop and Climate Sciences, University of the Free State, Bloemfontein, South Africa; 3 Science Advisory and Strategic Partnerships, Academy of Science of South Africa, Pretoria, South Africa; 4 Centre for Mineral Biogeochemistry, University of the Free State, Bloemfontein, South Africa; 5 Department of Chemistry, University of the Free State, Bloemfontein, South Africa; 6 Department of Microbiology and Biochemistry, University of the Free State, Bloemfontein, South Africa

**Keywords:** Heavy metals, Indian mustard, Phytoremediation, Contaminated water, Vetiver grass

## Abstract

One of the consequences of mining is the release of heavy metals into the environment, especially water bodies. Phytoremediation of areas contaminated by heavy metals using Vetiver grass and Indian mustard is cost-effective and environmentally friendly. This study aimed at enhancing remediation of heavy metal contaminated water through the simultaneous hybrid application of clay minerals (attapulgite and bentonite) and Vetiver grass or Indian mustard. A 21-day greenhouse experiment was carried out to investigate the effectiveness of the clay minerals to improve heavy metal phytoremediation. The highest accumulation of aluminium (Al) by Vetiver grass was 371.8 mg/kg in the BT2.5VT treatment, while for Mn, the highest accumulation of 34.71 mg/kg was observed in the AT1VT treatment. However, Indian mustard showed no significant uptake of heavy metals, but suffered heavy metal toxicity despite the addition of clay minerals. From this study, it was evident that bentonite added at 2.5% (w/v) could improve the phytoremediation capacity of Vetiver grass for Al and Mn polluted water. The current laboratory-scale findings provided a basis for field trials earmarked for remediation in a post-mining coal environment in South Africa. This remediation approach can also be adopted in other places.

## Introduction

Water is an important resource for sustenance, and has become a scarce resource in most parts of the world, thus demanding sustainable management. A major problem in water management is pollution, which hinders proper ecosystem functioning and diminishes its suitability for use in many sectors (Mahmoud et al. 2021). The rising human population drives industrialization, mining, agriculture, and poor sewage management, which then become major water pollutant sources (Danh et al. 2009; Mahmoud et al. 2022). Of the various contaminants, heavy metals are constantly released into the environment from a multitude of anthropogenic sources, posing a risk to human and environmental health (Danh et al. 2009; Isiuku and Enyoh 2019). The main sources of heavy metal pollutants are mining, manufacturing and processing industries, sewage, solid wastes, urban runoff and fuel leakages (WHO 2017). Humans can readily ingest heavy metals when they consume contaminated water and aquatic biota (Isiuku and Enyoh 2019).

South Africa is globally known as a key player in resource mining, which has resulted in many pollution problems despite the economic gains gathered from mining (Antin 2013). For example, The Leeuspruit River in proximity to a former coal mine records a concentration of 2.72 mg/L and 5.4 mg/L for Mn and Al respectively as against the acceptable limits of 0.1 mg/L and 0.5 mg/L for Al and Mn (Wessels 2013). The Leeuspruit River is a tributary of the Vaal River, which is considered to be South Africa’s key socio-economic surface water resource (DWAF 1996).

Although aluminum (Al) and manganese (Mn) are among the most abundant elements in the earth’s crust and are useful in various sectors (Wang et al. 2013), there is evidence that solubilized Al in toxic amounts negatively affects plants, animals and human beings. E.g., root growth inhibition in plants, nervous disorders and Alzheimer's disease in humans (Wang et al. 2013; Betancourt et al. 2015). Likewise, excess Mn levels in human beings can result in several health issues such as neurological disorders, low IQ in children and low coordination and movement control (Wang et al. 2013). In Ecuador, elevated levels of Mn (970 µg/L) were detected in the Puyango River and children in proximity to this river had over 2 μg/g in their hair. This was found to be responsible for neurobehavioral disorders and low IQ among these children (Betancourt et al. 2015).

Considering the negative impacts of Al and Mn on the environment, it is therefore important to ensure that the Leeuspruit River is brought to the best possible quality regulated by the South African water quality guidelines (DWAF 1996). One of the most accepted methods for the remediation of metals polluted media is phytoremediation, because it is affordable, and an easily applied green technology whose by-products can be used for other purposes such as bioenergy, essential oils and animal feed (Sricoth et al. 2018; Yang et al. 2019; Edgar et al. 2021). However, wider application of phytoremediation is inhibited by various challenges, including low biomass yield, extreme climatic influence, slow plant growth, long time required for remediation, pollutant-specific requirements, and adverse effects of contaminants on plant functions (Danh et al. 2009; Mioska 2012; Shahid et al. 2020; Leng et al. 2021; Sharma 2021).

Vetiver grass (*Chrysopogon zizanioides* L. Roberty) is notable in water remediation, because of its excellent physiological and morphological properties, which enable growth in contaminated substrates and harsh climatic conditions (Truong and Hartm 2001; Danh et al. 2009; Koupai et al. 2020). Kiiskila et al. (2019) observed that Vetiver reduced Ni, Zn, sulphate, Mn, Cr, Al and Cu by 38%, 35%, 28%, 27%, 21%, 11% and 8% respectively from acid mine drainage within a year. Indian mustard (*Brassica juncea* L. Czern) is also recognized as a good plant for phytoremediation (Qadir et al. 2004; Raj et al. 2020; Gravand and Rahnavard 2021), although field studies on water remediation by this plant are limited. Studies have confirmed the capacity of Indian mustard to survive and take up heavy metals with concentrations as high as 50 ppm in substrates (Meyers et al. 2008; Singh and Fulekar 2012; Napoli et al. 2019). Several Indian mustard genotypes were tested for their ability to accumulate Hg in a hydroponic experiment (Ansari et al. 2021). It was revealed that all the tested genotypes of Indian mustard could accumulate Hg. The observed accumulation of Hg in the roots of Indian mustard ranged from 18.4 to 269.9 μg/g. Singh and Fulekar (2012) observed that Indian mustard uptake was 25.000 μg/g, 32.750 μg/g and 30.550 μg/g of Cd, Pb and Zn from the soil, after 21 days of exposure.

Recently, clay minerals and nanoparticles have received attention for application in the remediation of contaminated soil and water (Otunola and Ololade 2020; Hussain et al. 2021). A review by Paz-Ferreiro et al. (2014) confirmed the use of several amendments such as biochar and compost in combination with phytoremediation plants to achieve better remediation results. The approach of using immobilisers to improve phytoremediation has been tested in the laboratory showing increased phytoextraction of Pb and Sb, up to 533 times higher than using plants alone without amendments (Katoh et al. 2016).

Approximately, not many studies have focused on the combined application of adsorbents and phytoremediation for metals-polluted water. Therefore, in pursuit of sustainable solutions to environmental pollution, this study evaluated the hybrid application of phytoremediation (using Vetiver grass and Indian mustard) and clay minerals (attapulgite and bentonite) for removing Al and Mn from polluted water. In particular, the study investigated the impact of attapulgite and bentonite at two dosage levels (1% and 2.5%) on the growth and metals removal potential of Vetiver grass and Indian mustard in water. This study determined the impact of attapulgite and bentonite on the phytoremediation capacity of Indian mustard and Vetiver grass in Al and Mn contaminated water. This study optimized the remediation of metals-polluted water through a hybrid application of phytoremediation and clay minerals. This application was undertaken to develop a suitable solution for the remediation of heavy metals in a post-mining environment in Sasolburg, South Africa. The findings of this study can also be applied in other areas.

## Materials and methods

### Water sampling

The study area is within a former coal mining area located in Sasolburg, Free State Province, South Africa. Mining operations were stopped in 2006 and the area is now at the rehabilitation and reclamation stage. Previous monitoring of this area established that the Leeuspruit River (26°50′16.1"S 27°48′42.3"E), one of the major water bodies in the area is polluted by nutrients and heavy metals including Al and Mn, emanating from mining as well as post-mining land-use activities (Wessels 2013).

To determine the heavy metal concentrations, water samples were collected in triplicate from four sites along the course of the river, based on land use patterns and suspected pollution sources. Physicochemical water parameters, including pH, temperature, electrical conductivity (EC), and total dissolved solids (TDS) were measured on-site using a calibrated standard multi-parameter probe (YSI Incorporated, Model 85D, I.N058500, SN 09 K 100,684, Yellow Springs, Ohio, USA). Clean 500 mL polyethylene bottles were rinsed three times with the river water before samples were collected and stored in cooler boxes with ice. The samples were transported to the Institute for Groundwater Studies at the University of the Free State for heavy metal and nutrient analyses.

### Plant preparation

Vetiver grass was supplied by Hydromulch (Pty) Ltd. Johannesburg, South Africa. The plants were thoroughly rinsed to remove soil particles and other possible contaminants. Indian mustard seeds were supplied by Seeds for Africa, South Africa. These were propagated in seedling trays using Hygrotech seedling starter composed of 17.2% N, 7.1% P, 2.3% K, 0.8% Ca, 0.2% Mg, 785 mg/kg Fe, 398 mg/kg Mn, Zn and Cu, 204 mg/kg B and 6.6 mg/kg Mo. The seedlings were kept moist in a greenhouse at the Department of Soil, Crop and Climate Sciences, University of the Free State. Thirty-day-old seedlings of similar sizes were thoroughly rinsed and used for the experiment.

### Properties of attapulgite and bentonite clays

Natural attapulgite and bentonite in their raw forms were supplied by AttaClay (Pty) Ltd (Germiston, South Africa). Brunauer–Emmett–Teller (BET) surface area and micropore of the clays were determined (Dogan et al. 2007). Cation exchange capacity (CEC), pH, EC, bulk density, water absorption and colour were also reported.

### Experiment set up

A 21-day randomized complete block design hydroponic experiment was set up in a greenhouse facility at the University of the Free State, South Africa. Experimental jars were maintained under temperatures of 28 °C (day) and 20 °C (night) and exposed to natural light. The treatment codes and descriptions are presented in Table 1 and there were three replicates of each treatment. This study seeks to determine the best hybrid of clay minerals and plants suitable for the remediation of Al and Mn contaminated water. Therefore, based on the highest concentrations found in the Leeuspruit River (Table 2), nutrient water (composed of water with a nutrient mixture of 9.9% N, 25.3% P, 47.7% K and 12.9% Mg) was spiked with Al (5 mg/l) and Mn (1 mg/l) using Al_2_(SO_4_)_3_ and MnCl_2_.4H_2_O reagents. The concentration of Al and Mn used in this experiment was based on the observed concentrations in the Leeuspruit River at the time of the experiment. Plastic jars of 1 L were used to hold 800 mL of contaminated water. These were covered with lids that had holes and wrapped with aluminium foil to minimize the effects of sunlight. The plants were placed in the water (one plant per jar) and the jars were refilled to the initial volume (800 mL) with more of the nutrient water each time the water levels were reduced through evaporation, and or transpiration consumption by the plants. Indian mustard seedlings with at least three leaves and a height of ~ 6 cm were selected for this experiment, while each Vetiver grass was trimmed to similar heights of 30 cm for shoots and 15 cm for roots before they were transplanted to individual jars.

**Table 1:** Water treatment codes and conditions

Treatment Code	Conditions
Control (Zero treatment)	Nutrient water only (with no added Al or Mn)
AT1	Attapulgite Applied at 1% (w/v)
AT2.5	Attapulgite Applied at 2.5% (w/v)
BT1	Bentonite applied at 1% (w/v)
BT2.5	Bentonite Applied at 2.5% (w/v)
VT	Vetiver only
BJ	Indian mustard only
AT1VT	Attapulgite applied at 1% (w/v) + Vetiver
AT2.5VT	Attapulgite applied at 2.5% (w/v) + Vetiver
BT1VT	Bentonite applied at 1% (w/v) + Vetiver
BT2.5VT	Bentonite applied at 1% (w/v) + Vetiver
AT1BJ	Attapulgite applied at 1% (w/v) + Indian mustard
AT2.5BJ	Attapulgite applied at 2.5% (w/v) + Indian mustard
BT1BJ	Bentonite applied at 1% (w/v) + Indian mustard
BT2.5BJ	Bentonite applied at 2.5% (w/v) + Indian mustard
VTC	Vetiver only in nutrient water
BJC	Indian mustard only in nutrient water

**Table 2:** Physicochemical parameters and heavy metals (± standard deviation) measured in-situ and laboratory chemical analysis of water samples from the Leeuspruit Assessment against the In‐stream Water Quality Guidelines (WQG) for the Leeuspruit Catchment

Sampling site	pH	Temperature (°C)	EC (mS/m)	TDS (mg/L)	NO_3_^−^(mg/L)	PO_4_^3−^ (mg/L)	SO_4_^2−^ (mg/L)	Al (mg/L)	Mn (mg/L)
RIVC	6.02 ± 0.01	25.5 ± 0.40	39.3 ± 0.50	240 ± 0.70	0.06 ± 0.01	0.24 ± 0.35	13.7 ± 0.41	0.96 ± 0.01	0.54 ± 0.001
RIV1	6.00 ± 0.00	26.7 ± 0.10	61.9 ± 0.10	364 ± 0.58	0.41 ± 0.02	0.32 ± 0.02	62.6 ± 0.77	4.58 ± 0.00	0.14 ± 0.00
RIV2	7.22 ± 0.07	29.7 ± 0.10	59.9 ± 0.10	965 ± 0.00	0.56 ± 0.02	0.51 ± 0.02	238.7 ± 1.44	0.64 ± 0.00	0.26 ± 0.00
RIV3	6.63 ± 0.10	25.0 ± 0.20	255.5 ± 1.30	580 ± 2.60	2.09 ± 0.09	2.06 ± 0.61	49.9 ± 0.53	0.33 ± 0.01	0.14 ± 0.00
WQG for the Leeuspruit Catchment	6–8.5	–	< 45.00	–	0.50	0.20	–	0.30	< 8.00

*EC* electrical conductivity (mS/m); *TDS* total dissolved solids

### Plant harvesting and processing

At the end of 21 days, the plants were harvested and carefully rinsed with water. The length and weight of the roots and shoots of the plants’ fresh biomass were recorded. The plant parts (roots and shoots) were then separately oven-dried at 75 °C for 72 h. The dry biomass of each plant was weighed, recorded and the tolerance index (TI) was calculated according to Nabaei and Amooaghaie (2020) as

1 $$\mathrm{TI}=\mathrm{Dry}\;\mathrm{biomass}\;\mathrm{in}\;\mathrm{contaminated}\;\mathrm{water}/\mathrm{Dry}\;\mathrm{biomass}\;\mathrm{in}\;\mathrm{uncontaminated}\;\mathrm{water}$$

The translocation factor (TF), which is the ability of a plant to translocate metals from its roots to shoot was calculated according to Nabaei and Amooaghaie (2020):

2 $$\mathrm{TF}=\mathrm{Heavy}\;\mathrm{metal}\;\mathrm{concentration}\;\mathrm{in}\;\mathrm{the}\;\mathrm{shoot}/\mathrm{Heavy}\;\mathrm{metal}\;\mathrm{concentration}\;\mathrm{in}\;\mathrm{the}\;\mathrm{root}$$

### Plant sample digestion and analysis

The dried root and shoot samples were milled and digested using microwave-assisted digestion by nitric acid. The homogenized powdered plant sample was weighed (0.5 ± 0.005 g), digested using nitric acid (HNO_3_) in a microwave and analyzed for heavy metals using Prodigy7 inductively coupled plasma–optical emission spectrometry (ICP-OES) (Teledyne Leeman Labs) at the Analytical Laboratory, Chemistry Department, University of the Free State ICP-OES (Sastre et al. 2002; US EPA 2007).

### Statistical analysis

All data were subjected to statistical analysis and expressed as means ± standard deviation of three replicates using analysis of variance (ANOVA). Means were considered significant at *ρ* < *0.05.* The calculations were performed using R software version 4.0.0 (R Development Core Team 2020). The mean values were compared using the ANOVA test for normal data, after which a Tukey’s post hoc test was performed to know the specific treatments with significant differences.

## Results

This research aimed at investigating the potential of Vetiver grass and Indian mustard with attapulgite and bentonite for effective removal of Al and Mn from contaminated water. This was observed in a 21-day greenhouse experiment, guided by the actual heavy metals concentration in the Leeuspruit River at the time of the experiment. The findings of this study are presented in the following section.

### Physicochemical properties and heavy metals

The physicochemical properties and heavy metal contents of the water samples for each sampling site along the Leeuspruit River were compared with the In‐stream Water Quality Guidelines for the Leeuspruit Catchment (In-stream WQG 2021). The results are presented in Table 2.

### Properties of attapulgite and bentonite clays

The clay minerals have a high specific surface area (SSA) and cation exchange capacity (CEC) (Table 3). Both attapulgite and bentonite have the same pH of 8.6 which is alkaline, thus encouraging metal immobilization. Other properties of attapulgite and bentonite are shown in Table 3.

**Table 3:** Properties of attapulgite and bentonite used in this study

Properties	Attapulgite	Bentonite
Specific surface area (SSA)	129.42 m^2^/g	111.16 m^2^/g
Micropore spaces	0.014 cm^3^/g	0.022 cm^3^/g
Cation exchange capacity (CEC)	73.90 meq/100 g	78.23 meq/100 g
pH	8.6	8.6
Electrical conductivity (EC)	52	71
Bulk density	1 ton/m3	1.1 ton/m3
Water absorption	193%	143%
Colour	Light grey	Brown

### Tolerance index (TI) and visual symptoms

**Fig. 1:**
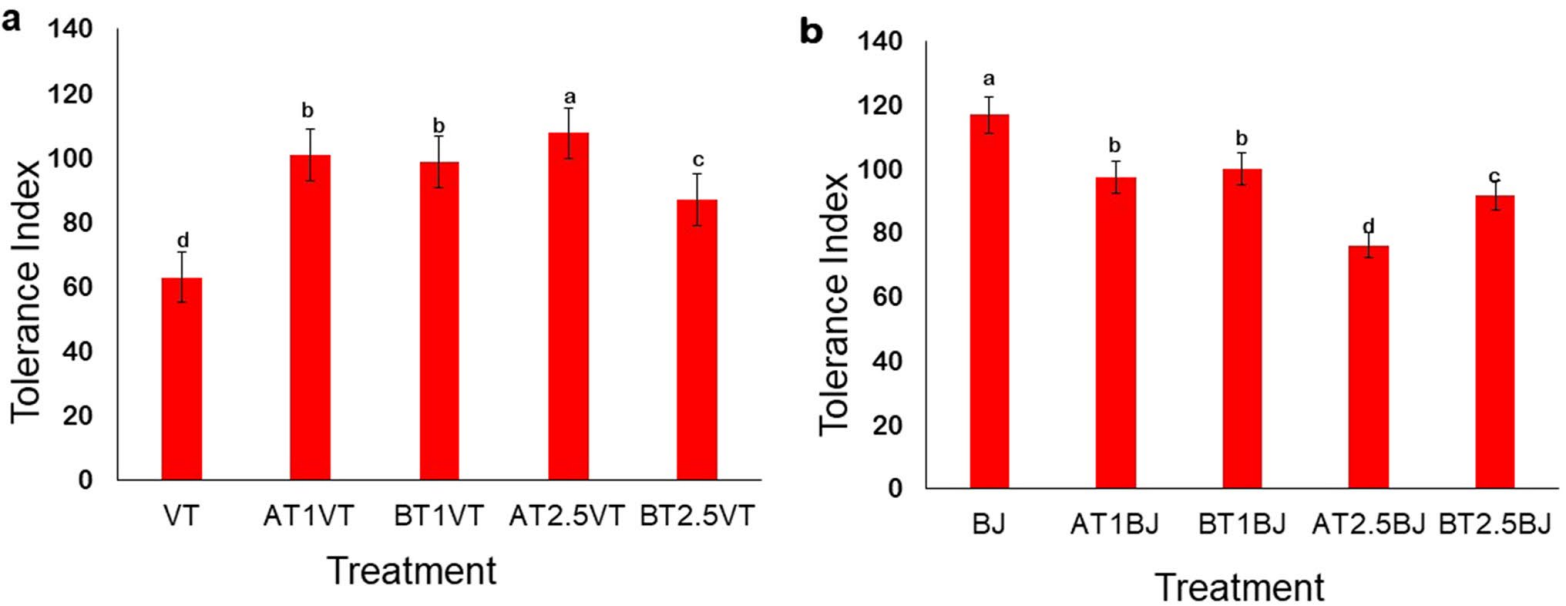
Tolerance index (TI) of (**a**) Vetiver grass and (**b**) Indian mustard in the various treatments at the end of the experiment. Data shown as mean ± SD of triplicates (Error bars represent standard deviation). Lower case letters on top of error bars indicate statistically significant variance between means. Key: VT- Vetiver grass only; AT1VT- Vetiver + attapulgite applied at 1% (w/w); AT2.5VT- Vetiver + attapulgite applied at 2.5% (w/w); BT1VT- Vetiver + bentonite applied at 1% (w/w); BT2.5VT- Vetiver + bentonite applied at 2.5% (w/w); BJ- Indian mustard only; AT1BJ- Indian mustard + attapulgite applied at 1% (w/w); AT2.5BJ- Indian mustard + attapulgite applied at 2.5% (w/w); BT1BJ- Indian mustard + bentonite applied at 1% (w/w); BT2.5BJ- Indian mustard + bentonite applied at 2.5% (w/w)

TI varied within the different treatments, but both plants showed a high tolerance with TI > 60 as shown in Figs 1a and b.

### Heavy-metal accumulation

**Fig. 2:**
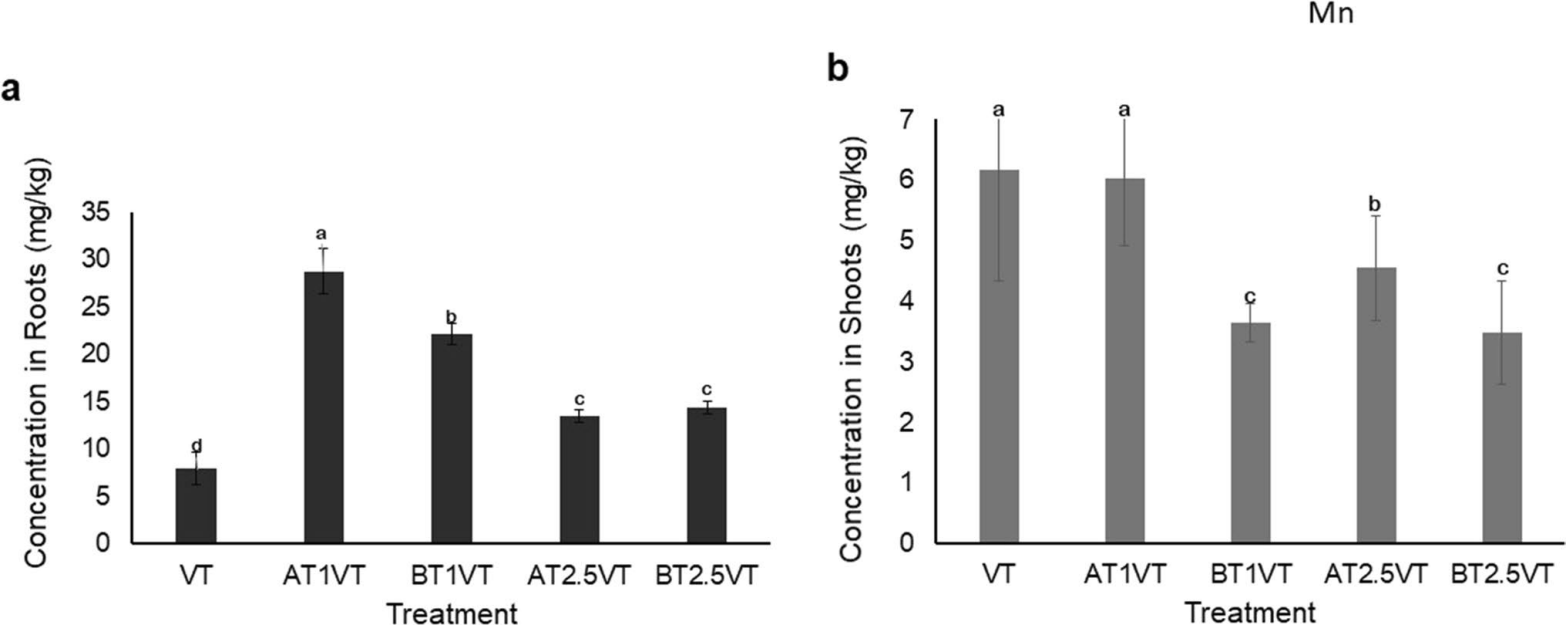
Concentrations of manganese (Mn) in (**a**) roots and (**b**) shoots of Vetiver grass in the different treatments at the end of the experiment. Data shown as mean ± SD of triplicates (Error bars represent standard deviation). Lower case letters on top of error bars indicate statistically significant variance between means (*ρ* < 0.05) based on Tukey’s Honest Significant Difference (HSD) test. Data with non-significant variance have the same letter. Key: VT: Vetiver grass only; AT1VT: Vetiver + attapulgite (1% w/v); AT2.5VT: Vetiver + attapulgite (2.5% w/v); BT1VT: Vetiver + bentonite (1% w/v); BT2.5VT: Vetiver + bentonite (2.5% w/v)

**Fig. 3:**
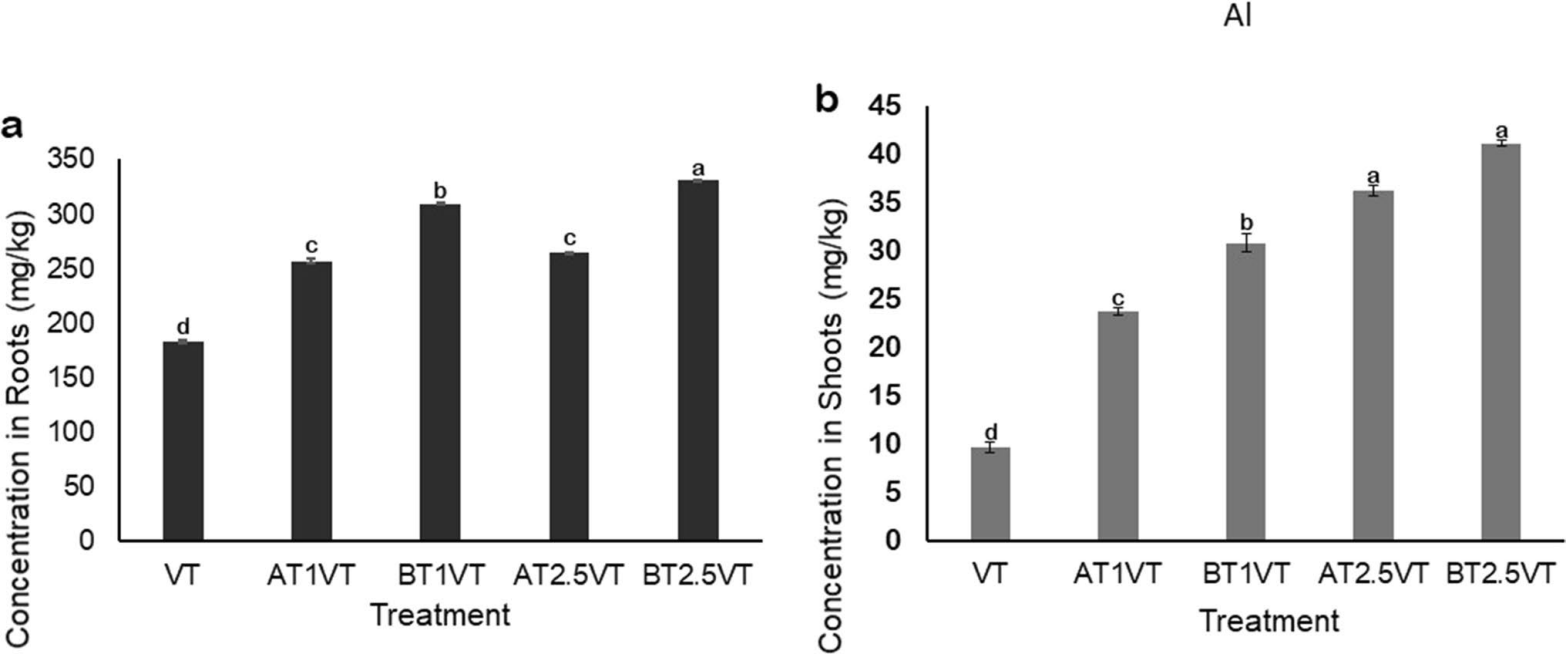
Concentrations of aluminum (Al) in (**a**) roots and (**b**) shoots of Vetiver grass in the different treatments at the end of the experiment. Data shown as mean ± SD of triplicates (Error bars represent standard deviation). Lower case letters on top of error bars indicate statistically significant variance between means (*ρ* < 0.05) based on Tukey’s Honest Significant Difference (HSD) test. Data with non-significant variance have the same letter. Key: VT: Vetiver grass only; AT1VT: Vetiver + attapulgite (1% w/v); AT2.5VT: Vetiver + attapulgite (2.5% w/v); BT1VT: Vetiver + bentonite (1% w/v); BT2.5VT: Vetiver + bentonite (2.5% w/v)

The accumulated Al and Mn in the roots and shoots of Vetiver grass in the various treatments are shown in Figs. 2 and 3. The concentration of Al and Mn left in the treated water at the end of the experiment was determined and presented in Table 4. The metal concentration was reduced in the vegetated treatments compared to the unvegetated treatments.

**Table 4:** Residual Al and Mn contents in treated water under various treatment conditions

Heavy metals	OT (mg/l)	BT1 (mg/l)	BT1VT (mg/l)	AT2.5VT (mg/l)	BT2.5VT (mg/l)	BT2.5 (mg/l)	VT (mg/l)	AT1VT (mg/l)	AT2.5 (mg/l)	AT1 (mg/l)
Mn	24.5 ± 8.21	13 ± 7.85	0.1 ± 0.11	1.6 ± 1.02	0.5 ± 0.26	2.9 ± 0.67	16.8 ± 3.82	3.8 ± 3.30	0.7 ± 0.08	15.1 ± 1.44
Al	39.3 ± 15.93	30.9 ± 23.53	0.7 ± 0.61	3.4 ± 6.01	2.9 ± 2.44	17.5 ± 2.46	15.9 ± 5.42	14.5 ± 19.22	1.2 ± 0.08	2.1± 0.42

Values are Means ± standard deviations (*n* = 3), *p* < 0.05. Key: OT- zero treatment; VT- Vetiver grass only; AT1VT- Vetiver + attapulgite (1% w/v); AT2.5VT- Vetiver + attapulgite (2.5% w/v); BT1VT- Vetiver + bentonite (1% w/v); BT2.5VT- Vetiver + bentonite (2.5% w/v); AT1- attapulgite (1% w/v); AT2.5- attapulgite (2.5% w/v); BT1- bentonite (1% w/v); BT2.5- bentonite (2.5% w/v)

### Translocation factor (TF)

The TF for Al and Mn in all the treatments was less than 1 as presented in Table 5. The VT treatment (Vetiver grass alone) showed the highest TF for Al, but the same treatment showed the lowest TF for Mn.

**Table 5:** Translocation factor (TF) observed for Vetiver grass in each treatment

Heavy Metal	BT2.5VT	AT1VT	AT2.5VT	VT	BT1VT
Mn	0.242	0.210	0.337	0.776	0.165
Al	0.124	0.092	0.137	0.052	0.104

Values are Means ± standard deviations (*n* = 3). (*p* < 0.05), a strong negative correlation was observed between the TF of Al and Mn. Coefficient (r) = -0,68. Key: VT- Vetiver grass only; AT1VT- Vetiver + attapulgite (1% w/v); AT2.5VT- Vetiver + attapulgite (2.5% w/v); BT1VT- Vetiver + bentonite (1% w/v); BT2.5VT- Vetiver + bentonite (2.5% w/v)

### Principal component analysis (PCA)

**Fig. 4:**
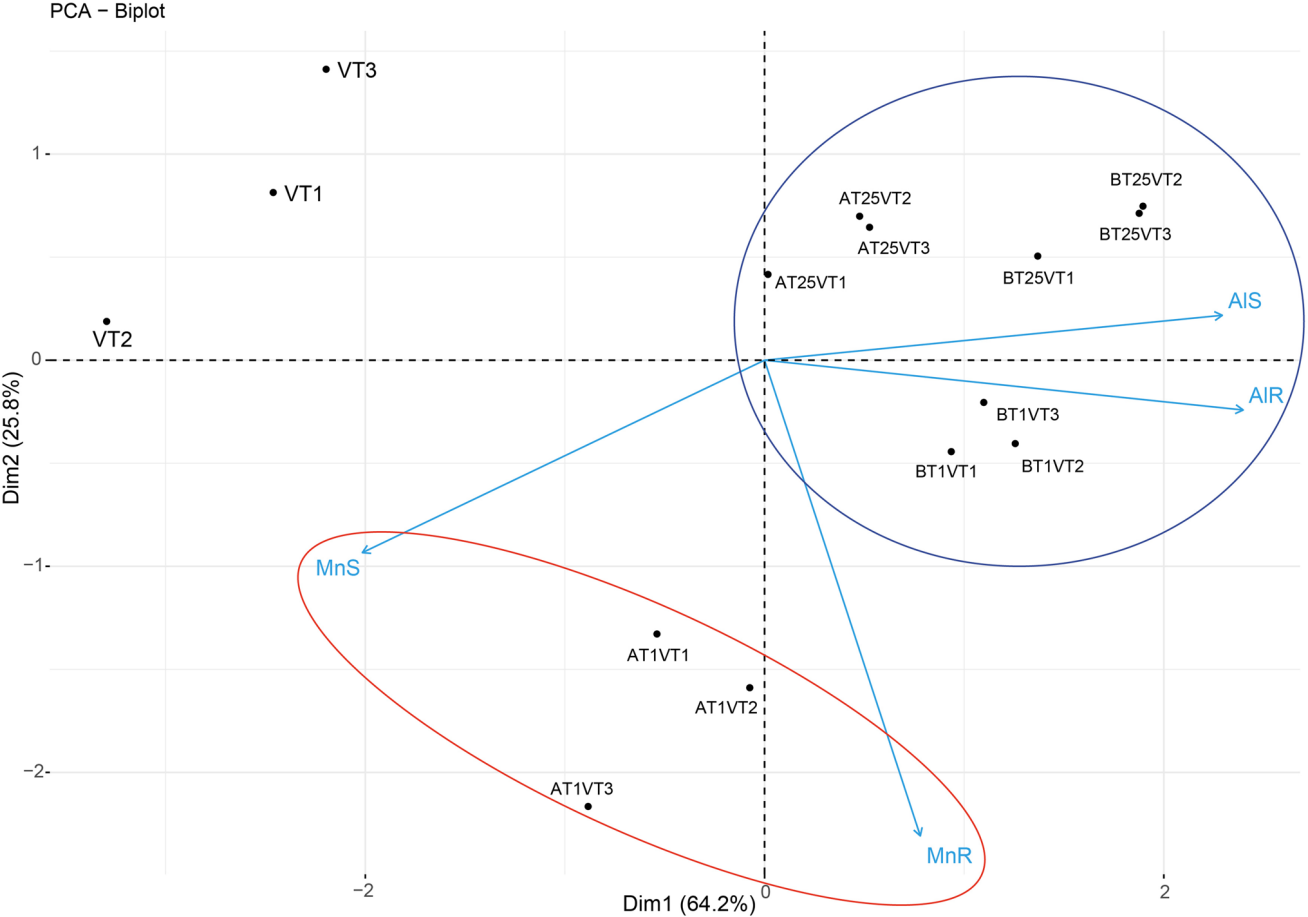
Principal component analysis (PCA) for the accumulation of Al and Mn in the different treatments. Key: VT: Vetiver grass only; AT1VT: Vetiver + attapulgite (1% w/v); AT2.5VT: Vetiver + attapulgite (2.5% w/v); BT1VT: Vetiver + bentonite (1% w/v); BT2.5VT: Vetiver + bentonite (2.5% w/v)

The PCA biplot grouped the treatments into three as shown in Fig. 4, indicating a negative correlation between the groups.

## Discussion

The pH values observed for the water samples indicated a close to neutral pH for the Leeuspruit. The measured temperature and pH were within the Leeuspruit Catchment Water Quality Limits. There was a high variation in the TDS values among the sample points, which ranged from 240 ± 0.70 mg/L to 965 ± 0 mg/L. The possible sources of the dissolved solids may have been the dissolution of underlying sedimentary rocks or runoff from agricultural land (Fondriest Environmental 2014). Based on the Leeuspruit Catchment Water Quality Guidelines, ideal EC values were exceeded in all the sites except for RIVC (Table 2). The highest PO_4_^3−^ value of 2.06 ± 0.61 mg/L was from the sampling point RIV4, which exceeded this limit by 1.66 mg/L. High phosphate levels can promote eutrophication, lowering overall water quality (Mezgebe et al. 2015). The SO_4_^2−^ concentrations ranged from 13.7 ± 0.41 mg/L to 238.7 ± 1.44 mg/L (RIV3), which indicates mine water pollution. The ICP-OES results revealed that Mn ranged from 0.14 ± 0.00 to 0.54 ± 0.00 mg/L while Al ranged from 0.33 ± 0.01 to 4.58 ± 0.00 mg/L. The result for other heavy metals such as Cd, As, Co, Cr, Mo, and Cu was below detection limits. Al and Mn with concentrations of ~ 5 mg/L and 1 mg/L respectively were of importance in this research.

SSA and CEC determine the adsorptive properties of clay minerals (Macht et al. 2011), while the total negative charges available in the clays enable them to hold heavy metals unto their surfaces. The SSA observed indicates the availability of internal and external layers within attapulgite and bentonite that are available as reactive surfaces (Otunola and Ololade 2020). An increase in pH increases the adsorptive ability of the clays, therefore, the observed pH of both clays further indicates their capability for adsorption of Al and Mn in polluted water.

The rate of phytoremediation is affected by the plant growth rate, which is why fast-growing and high-biomass crops are the most appropriate (Danh et al. 2009; Isiuku and Enyoh 2019; Itam et al. 2019). The TI of Vetiver grass was significantly higher in the treatments assisted with attapulgite and bentonite. Attapulgite alleviated heavy metals stress and gave the best TI of 107.7 in the AT2.5VT treatment, while the lowest TI of 62.9% was obtained in the Vetiver grass + control (Fig. 1a) because it experienced greater metal stress. This indicates that attapulgite and bentonite alleviated heavy metal stress in Vetiver grass. For the Indian mustard treatments, the highest TI was 116.8 with the BJ treatment (Indian mustard + control), while the lowest TI of 76.2 was observed in the AT2.5BJ treatment (Fig. 1b), indicating that the clay minerals did not improve the metal tolerance of Indian mustard. Despite the addition of more contaminated water leading to increased concentrations of Al and Mn, Vetiver grass showed no physical signs of heavy metal stress. The plant remained green and luscious throughout the experiment, while Indian mustard became pale and yellowish with leaves drying out due to heavy metal stress. This confirms that Vetiver can survive in highly contaminated environments (Danh et al. 2009; Suelee et al. 2017). This is similar to the observation of Gravand and Rahnavard (2021), who noted that there were no physical signs of toxicity in Vetiver grass in highly contaminated media. The variation in the TI of Vetiver grass and Indian mustard may be due to the plants’ different morphological and cellular traits (Isiuku and Enyoh 2019). Attapulgite and bentonite did not necessarily favour an increase in heavy metal tolerance and growth of Indian mustard, but they successfully improved the tolerance of Vetiver grass in Al and Mn contaminated water.

At the end of the experiment, the concentration of Al and Mn was reduced in the vegetated treatments compared to the unvegetated treatments (Table 4). The treatments comprising both clay minerals and plants showed higher heavy metal removal. This was because more contaminated water was added to the initial 800 mL mark as the water evaporated or transpired, leading to an increasing concentration of heavy metals available for uptake.

The desired outcome was to determine the effects of attapulgite and bentonite on the tolerance and heavy metal accumulation of Vetiver grass and Indian mustard. The experiment revealed the quantity of Al and Mn that the plants could take up in 21 days under different treatments. For the clay-only treatments, there may have been a regeneration of contaminants that were previously adsorbed (Li et al. 2019; Said et al. 2020). This explains why Al and Mn in some treatments increased, although the heavy metals were expected to have been adsorbed by the clay minerals in the clay-only treatments (Table 4).

A statistically significant difference (*p* = 0.014) was recorded in the concentrations of Al and Mn between the treatments, as well as in their roots and shoots (Figs. 3 and 4). There was generally higher root uptake of both heavy metals in all the treatments. The significant variance in root and shoot uptake corresponds to the findings of previous studies and confirms that Vetiver accumulates most heavy metals in its roots, because of its high tolerance (Suelee et al. 2017; Hassan et al. 2020; Gravand and Rahnavard 2021). This was confirmed in all the treatments with Vetiver, even those that were assisted with attapulgite and bentonite.

The plant uptake of Al (Fig. 3) was generally higher than that of Mn (Fig. 2) in both roots and shoots, probably because the initial concentration of Al in the water was five times greater than that of Mn, and in most cases, heavy metal accumulation in plants increases with increasing concentration in the substrates (He et al. 2021; Hussain et al. 2021; Leng et al. 2021). In addition, Al could have reduced the availability of Mn because it exhibits an antagonistic behavior towards Mn uptake (Yang et al. 2009). Al has more affinity to FeOH than Mn, therefore the iron plaques might also play an important role in this process. In previous studies, Vetiver showed a preference for Mn over other heavy metals, without a significant change in biomass yield even at high concentrations (Hassan et al. 2020; Thakur et al. 2021). For Mn, AT1VT showed the highest root uptake (28.68 ± 1.95 mg/kg) while the VT (Vetiver only treatment) showed the least root uptake (7.9 ± 1.38 mg/kg). The highest shoot uptake of 6.1 ± 1.49 mg/kg was, however, observed in the VT treatment. According to Thakur et al. (2021), when heavy metals are taken up into plant cells, they can be excluded, immobilized, chelated, or compartmentalized. Therefore, cell growth determines biomass yield, which in turn promotes metal uptake (Ali et al. 2013). For Al, treatment BT2.5VT showed the highest root and shoot uptake of 330.7 ± 0.47 mg/kg and 41.1 ± 0.22 mg/kg, respectively, while the lowest root and shoot uptake was observed in treatment VT. There was a strong positive correlation between root and shoot Al uptake by Vetiver grass (r = 0.90, *p* < *0.05*), while a weak positive coefficient was observed between Mn root and shoot uptake (r = 0.01, *p* < *0.05*).

According to the PCA biplot (Fig. 4), three groups are observed consisting of samples without clay amendment (VT samples), Samples with 1% of clay materials (AT1VT and BT1VT samples), and those with 2.5% clay (AT2.5VT and BT2.5VT), indicating the negative correlation between the groups. Also, it is observed that Al accumulated in the roots and shoots is associated mainly with the BT1VT, AT2.5VT, and BT2.5VT experiments, while the Mn accumulated in the roots and shoots is associated mainly with AT1VT. In addition, the PCA confirms the antagonistic behaviour towards Mn and Al uptake (Yang et al. 2009). However, it can be seen that the Al uptake is similar in both the roots and the shoots, while a statistically significant difference is observed between the uptake of Mn in shoots and roots.

Generally, there was no significant uptake of Al and Mn by Indian mustard in all treatments, as none of the heavy metals were detected by ICP-OES. This was attributed to the increasing concentrations of contaminated water in the experimental jars. Even in the untreated water, the final concentration for Mn and Al was 25.4 ± 8.21 and 39.3 ± 15.93 mg/L respectively (Table 4). These final concentrations resulted from the continual addition of contaminated water each time the initial volume was reduced by evaporation (in the unvegetated jars) and or transpiration and plant uptake (in the vegetated jars). Phytoremediation studies indicate that this method is suitable for minimally contaminated sites (Isiuku and Enyoh 2019). Although none of the plants died during the experiment, the resulting toxicity from increasing heavy metal concentration was likely to be responsible for the insignificant uptake of Al and Mn in Indian mustard.

Previous studies indicated that Indian mustard can uptake high concentrations of metals (50–30,000 ppm) in water (Meyers et al. 2008; Singh and Fulekar 2012; Napoli et al. 2019). However, studies have also indicated that Indian mustard performs better as a phytoremediation plant when only one metal type is present compared to when two or more contaminant or heavy metal types are present. For example, Yang et al. (2021) reported that Indian mustard performed better as a hyperaccumulator when only As or Pb was present compared to when both heavy metals were present. The authors noted up to a 90% decrease in As uptake when Pb was present as a co-contaminant in solution, whereas, in As only solution, uptake by Indian mustard was 1,786 ppm. Kim et al. (2010) observed a reduced uptake of Cd, Cu, Pb, and Zn due to the presence of multiple metals and the competitive uptake of these metals.

The insignificant metal uptake by Indian mustard could also result from Mn-induced toxicity, which has been reported previously (Parashar et al. 2014; Fariduddin et al. 2015). From these studies, it was evident that excess Mn triggers reactive oxidative stress such as H_2_O_2_ and O_2_ radicals in Indian mustard, threatening proper plant growth after damage to membrane lipids, stomatal functions, proteins, and enzymes (Parashar et al. 2014). According to Gayatri et al. (2019), higher contents of trace elements including Zn, Ni, Mn, Cu and Fe can inhibit plant growth and lead to toxicity in plants. This is likely to be the case with Indian mustard in this study as the increasing concentrations of Al and Mn may have lowered the ability of Indian mustard cells to function properly, thereby limiting its metabolic, morphological and absorptive properties (Srivastava et al. 2015; Phusantisampan et al. 2016). Mn and Al induced oxidative stress in Indian mustard, restrict plant growth, cell elongation and photosynthesis, leading to stunting (Fariduddin et al. 2015; Ahmad et al. 2018). In this study, attapulgite and bentonite could not increase heavy metal uptake by Indian mustard, and neither could these clay minerals alleviate heavy metal stress in the plant. Vetiver grass under the same experimental condition as Indian mustard exhibited more resistance to Al and Mn compared to Indian mustard.

Translocation factor values < 1 indicate a plant is suitable for phytostabilisation or root storage of heavy metals, and TF values > 1 indicate suitability for phytoextraction (Isiuku and Enyoh 2019). Mn showed a higher translocation to the shoots of Vetiver compared to Al in all the treatments with Vetiver grass (Table 5). The highest TF of 0.78 was observed in the VT treatment for Mn, indicating that attapulgite and bentonite might have prevented the translocation of Mn by promoting stronger adsorption of Mn within the root zone. According to Ramos-Arcos et al. (2019), the removal of Mn was the fastest among heavy metals including Al, B, Ba, Be, Co, Cr, Cu, Fe, Mg, Ni, Pb, S, Se, Tl, V and Zn, but TF was < 1. This is similar to the present study as TF values below 1 (ranging between 0.22 to 0.77) were observed for Mn in Vetiver grass. Another study showed that within 30 days, 0.15 ppm of Mn can be removed from landfill leachate by Vetiver grass (Thakur et al. 2021), with TF > 1. The high TF observed in the study may have been due to the low initial concentration of Mn, which encouraged faster translocation (Thakur et al. 2021). For Al, the highest TF of ~ 0.14 was observed in the AT2.5VT treatment (Table 5), but reasonable amounts of Al were stored within the roots of Vetiver. Generally, the results indicated that the roots of Vetiver grass could both tolerate and accumulate high concentrations of Mn and Al.

The low TF observed in this study was similar to the findings of Suelee et al. (2017) and Thakur et al. (2021). The cell membrane is negatively charged; therefore, Mn and Al ions enter plant cells easily. However, Mn is more easily translocated to the shoots because it is an essential element for plant growth (Ramos-Arcos et al. 2019; Shahid et al. 2020; Thakur et al. 2021). Although the TF indicates a plant’s ability to translocate heavy metals to its shoots, it should not be solely considered when determining the suitability of plants as hyperaccumulators, because although TF < 1, the shoots may still have high uptake levels of heavy metals. For instance, in a study on the uptake of Cd by Himalayan balsam, TF was < 1, but the plant’s shoots contained about 70% of the total Cd root uptake (Coakley et al. 2019). The TF was < 1 for Al and Mn in this study, a situation that can be considered an advantage, because it prevents metals from reaching the plant shoots and damaging the photosynthetic machinery as well as limiting post-remediation use of Vetiver grass (Isiuku and Enyoh 2019). This also reduces the amount of heavy metals introduced into the food chain because animals prefer to graze on other grass types than Vetiver grass due to the sharp edges on its leaf lamina (Truong and Hartm 2001).

## Conclusion

The findings of this study indicate that Vetiver grass is preferable for phytoremediation of Al and Mn, based on the fact that within 21 days, Vetiver grass could bioaccumulate significant amounts of Al and Mn, while Indian mustard could not uptake significant amounts of Al and Mn that could be detected by ICP-OES analysis. This study encourages the application of clay minerals such as attapulgite and bentonite to increase the heavy metal phytoremediation potential of Vetiver grass. These results suggest that Vetiver grass can be a suitable candidate for the removal of Al and Mn in contaminated water under controlled greenhouse conditions. Therefore, it is recommended that the efficacy of this combination of Vetiver grass and clay minerals be tested under field (natural) conditions to ascertain full-scale application for heavy metal-contaminated waters.

## Data Availability

The authors confirm that the data supporting the findings of this study are available within this published article.
